# Functional and expression analyses of two kinds of betaine aldehyde dehydrogenases in a glycinebetaine-hyperaccumulating graminaceous halophyte, *Leymus chinensis*

**DOI:** 10.1186/s40064-015-0997-4

**Published:** 2015-04-30

**Authors:** Shiro Mitsuya, Asumi Tsuchiya, Keiko Kono-Ozaki, Takashi Fujiwara, Teruhiro Takabe, Tetsuko Takabe

**Affiliations:** Graduate School of Bioagricultural Sciences, Nagoya University, Chikusa, Nagoya, 464-8601 Japan; Research Institute, Meijo University, Tenpaku, Nagoya, 464-8502 Japan

**Keywords:** Barley, Betaine aldehyde dehydrogenase, Choline monooxygenase, Enzyme kinetics, Salinity stress, Subcellular localization

## Abstract

Glycinebetaine (GB) is an important compatible solute for salinity tolerance in many plants. In this study, we analyzed the enzymatic activity and the expression level of betaine aldehyde dehydrogenase (BADH), an important enzyme that catalyzes the last step in the GB synthesis in *Leymus chinensis*, a GB-hyperaccumulating graminaceous halophyte, and compared with those of barley, a graminaceous glycophyte. We have isolated cDNAs for two *BADH* genes, *LcBADH1* and *LcBADH2*. LcBADH1 has a putative peroxisomal signal peptide (PTS1) at its C-terminus, while LcBADH2 does not have any typical signal peptide. Using immunofluorescent labeling, we showed that BADH proteins were localized to the cytosol and dot-shaped organelles in the mesophyll and bundle sheath cells of *L.chinensis* leaves. The affinity of recombinant LcBADH2 for betaine aldehyde was comparable to other plant BADHs, whereas recombinant LcBADH1 showed extremely low affinity for betaine aldehyde, indicating that LcBADH2 plays a major role in GB synthesis in *L. chinensis*. In addition, the recombinant LcBADH2 protein was tolerant to NaCl whereas LcBADH1 wasn’t. The kinetics, subcellular and tissue localization of BADH proteins were comparable between *L. chinensis* and barley. The activity and expression level of BADH proteins were higher in *L. chinensis* compared with barley under both normal and salinized conditions, which may be related to the significant difference in the amount of GB accumulation between two plants.

**Electronic supplementary material:**

The online version of this article (doi:10.1186/s40064-015-0997-4) contains supplementary material, which is available to authorized users.

## Background

Salinity is one of the biggest factors which limits the productivity of crops (Boyer 1982). In order to survive and continue growth, plants have developed many defense mechanisms which allow them to adapt to unsuitable environments. One of these mechanisms is the accumulation of compatible solutes. Compatible solutes differ among plant species and include sugar alcohols, amino acids and their derivatives, tertiary sulphonium compounds and quaternary ammonium compounds (Rhodes & Hanson 1993; Bohnert & Jensen 1996). Glycinebetaine (GB) is one of the most important compatible solutes. GB is present in bacteria, cyanobacteria, animals, and several plant families such as Graminaceae, Amaranthaceae, Asteraceae and Malvaceae (Rhodes & Hanson 1993), and is known to efficiently stabilize the structure and function of proteins (Papageorgiou & Murata 1995; Takabe et al. 1998) and to decrease the *T*_m_ of double-stranded DNA (Rees et al. 1993).

The biological effects of GB on plant stress tolerance have been shown using near-isogenic lines in maize and transgenic plants (Saneoka et al. 1995; Yang et al. 1995; Nomura et al. 1995, 1998; Sakamoto et al. 1998; Mohanty et al. 2002). In near-isogenic lines of maize differing in levels of GB accumulation, lines with abundant GB showed higher salt tolerance than GB-deficient lines (Saneoka et al. 1995; Yang et al. 1995). However, most crop plants such as rice accumulate little GB and are sensitive to salt stress. Therefore, to improve plant salt tolerance, it is important to elucidate the mechanism of GB biosynthesis to potentially introduce the ability of GB production into GB-nonaccumulators.

In plants, GB is synthesized by the oxidation of choline via a two-step process: choline → betaine aldehyde → GB (Rathinasabapathi et al. 1997). The first and second steps are catalyzed by choline monooxygenase (CMO) and betaine aldehyde dehydrogenase (BADH), respectively (Arakawa et al. 1987; Brouquisse et al. 1989; Wood et al. 1996; Rathinasabapathi et al. 1997; Hibino et al. 2001; Nakamura et al. 2001; Mitsuya et al. 2011). Both steps occur in chloroplasts in Amaranthaceae (Weigel et al. 1988). On the other hand, in barley, GB is produced in a cooperative way in peroxisomes and cytosol catalyzed by NADPH-dependent peroxisomal CMO (HvCMO) and cytosolic BADH (BBD2) (Fujiwara et al. 2008; Mitsuya et al. 2011), whereas it is not clear whether other graminaceous plants also have a non-chloroplastic pathway of GB production.

*Leymus chinensis* is a graminaceous and perennial grass that grows in the meadow steppes of Northeast China and Inner Mongolia, and is an important grass species for grazing (Kawanabe et al. 1994). *L. chinensis* consists of two ecotypes, a green type and a gray type, which differ in leaf color and ear shape Kawanabe et al. (1996). The gray type of *L. chinensis* is adaptable to saline, drought and alkaline conditions in comparison with the green type (Kawanabe et al. 1996) and can survive and still develop stolons in the presence of 500 mM NaCl (Ochiai and Matoh 2001). Moreover, *L. chinensis* can accumulate large amounts of GB (93 mM) which accounts for 30% of the total solute concentration in leaves grown in 200 mM NaCl (Ochiai and Matoh 2001), indicating that *L. chinensis* is a GB-hyper-accumulating halophyte. However, the biosynthetic enzymes of GB and their regulation mechanism in *L. chinensis* have not been characterized thus far.

The objective of this study is to identify GB-biosynthetic BADH proteins in the graminaceous halophyte *L. chinensis* and to compare its characteristic with that of barley, a graminaceous glycophyte. For this purpose, we have isolated cDNAs for two kinds of *BADH* genes from *L. chinensis*, *LcBADH1* and *LcBADH2*. Using the recombinant BADH proteins of *L. chinensis*, we have determined their activity and stability to NaCl. The effect of salinity stress on the expression level of mRNA and protein of LcBADH was also investigated in *L. chinensis* plants. Also in this paper, we discuss the difference of the characteristic of BADH proteins between *L. chinensis* and barley, that may be related to the significant difference of the accumulation level of GB between two plants.

## Results

### Cloning of cDNAs for *LcBADH1* and *LcBADH2* genes in *L. chinensis*

**Figure 1:**
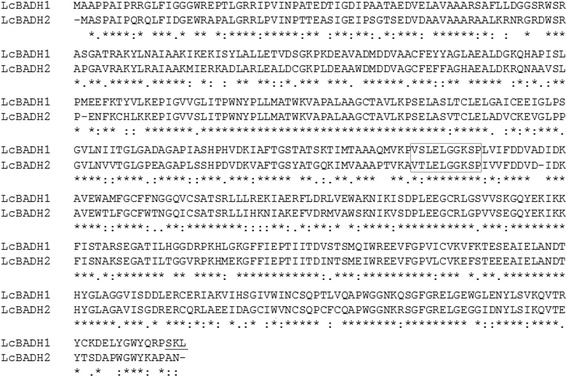
Comparison of the deduced amino acid sequences of the *LcBADH1* and *LcBADH2* genes. The deduced amino acid sequence of *LcBADH1* (GenBank Accession No. AB711137) was aligned with that of *LcBADH2* (BAD86758). The putative signal peptide for targeting to peroxisomes (SKL motif PTS1) is underlined. Ten amino acids boxed show a highly conserved region among aldehyde dehydrogenases. The symbols asterisk, colon and dot denote identical, conserved, and similar amino acids, respectively.

To isolate *BADH* cDNAs from *L. chinensis*, we constructed a cDNA library from leaves of salt-stressed *L. chinensis* and cloned *BADH* cDNAs by PCR using degenerate primers which were designed on the basis of the highly conserved amino acid sequences from several plants’ BADH as described in Methods. Next, by screening of a cDNA library using the *BADH* fragment, eleven clones were isolated and sequenced. The nucleotide sequences of two of the clones, and that of nine clones were the same and named *LcBADH1* and *LcBADH2*, respectively. The *LcBADH1* and *LcBADH2* cDNA contains an open reading frame of 1,521 bp and 1,509 bp encoding 506 and 502 amino acids, respectively (Figure 1). LcBADH1 has the putative peroxisomal signal peptide (Ser-Lys-Leu, underlined sequences in Figure 1) at its C-terminus (PTS1) (Baker and Sparkes 2005), while LcBADH2 does not have any typical signal peptide. Both *LcBADH1* and *LcBADH2* genes encode a conserved decapeptide (Val-Thr/Ser-Leu-Glu-Leu-Gly-Gly-Lys-Ser-xPro, xboxed sequences in Figure 1) which is highly conserved among general aldehyde dehydrogenases (Weretilnyk and Hanson 1990). LcBADH2 has cysteine at position 444 that is critical for high affinity to betaine aldehyde (Díaz-Sánchez et al. 2012) whereas LcBADH1 possesses a low betaine aldehyde affinity isoleucine at the corresponding position.

**Figure 2:**
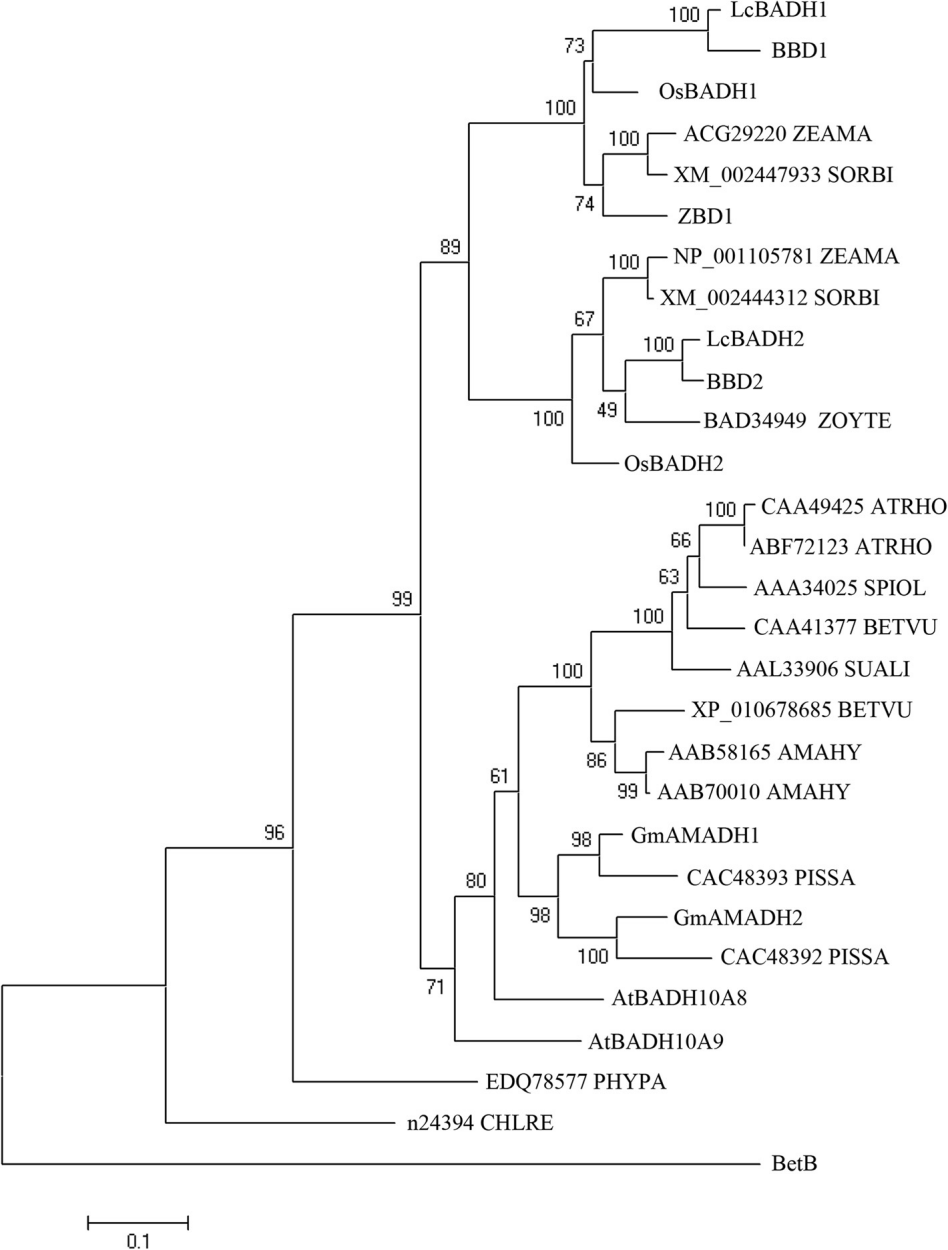
A phylogenetic tree of plant BADH proteins. Deduced polypeptide sequences included in this comparison are from *Leymus chinensis* (LcBADH1; GenBank Accession No. AB711137 and LcBADH2; BAD86758), barley (BBD1; BAA05466 and BBD2; BAB62846), rice (OsBADH1; BAA21098 and OsBADH2; BAC76608), sorghum (XM_002444312 and XM_002447933), *Zoysia tenuifolia* (ZBD1; BAD34957 and BAD34949), spinach (SoBADH; AAA34025), *Atriplex hortensis* (CAA49425 and ABF72123), sugar beet (CAA41377 and ZP_010678685), *Suaeda liaotungensis* (AAL33906), *Amaranthus hypochondriacus* (AAB58165 and AAB70010), *Glycine max* (GmAMADH1; BAG09377 and GmAMADH2; BAG09376), *Pisum sativum* (CAC48392 and CAC48393), *Arabidopsis thaliana* (AtALDH10A8; NM_106150 and AtALDH10A9; NM_114686), *Physcomitrella patens* (EDQ78577), *Chlamydomonas reinhardtii* (n24394) and *Escherichia coli* (BetB; AAA23506). Multiple sequence alignment and the generation of the phylogenetic tree were performed using ClustalX and MEGA6, respectively. The scale bar represents 0.1 substitutions per site. The numbers indicated for each node are the bootstrap values.

We performed a phylogenetic analysis using deduced polypeptide sequences of plant BADHs from six species of Graminaceae, five species of Amaranthaceae, two species of Fabaceae and *Arabidopsis thaliana* as a Brassicaceae. The result showed that plant BADHs were divided into two groups, monocotyledonous and dicotyledonous BADHs and that LcBADH1 and LcBADH2 were in the monocotyledonous BADH group (Figure 2). Furthermore the monocotyledonous BADH group was divided into two subgroups, which was consistent with the result of Arikit et al. (2011). The dicotyledonous BADH group was divided into amaranthaceous and fabaceous subgroups. LcBADH1 with the SKL motif was similar to peroxisomal barley BBD1 (93% similarity) whereas LcBADH2 with no typical signal peptide was highly homologous with cytosolic barley BBD2 (96% similarity) (Figure 2). Interestingly, most monocotyledonous BADHs, with the exception of LcBADH2 and BBD2, harbor an SKL motif at their C-terminus. Although it was reported that GB is synthesized in chloroplasts in plants (Chen and Murata 2011), graminaceous plants may have a different site for GB biosynthesis other than the chloroplasts.

### BADH activity of recombinant LcBADH1 and LcBADH2 proteins

To determine the enzymatic characteristics of LcBADH1 and LcBADH2 proteins, both proteins were expressed in *E. coli* and purified by affinity chromatography. After enterokinase treatment for the removal of pET32a vector-derived tags, the eluates were separated by SDS-PAGE, which showed that both enzymes were purified to apparent homogeneity (Additional file 1). The molecular mass of the purified proteins corresponded to that of the deduced amino acid sequences of LcBADH1 and LcBADH2 (approx 54 kDa).

Using these purified recombinant BADHs, we investigated the Michaelis constant (*K*_m_ value) of LcBADH1 and LcBADH2 for betaine aldehyde (Additional file 2). The *K*_m_ values of LcBADH1 and LcBADH2 were 27300 μM and 109 μM, respectively (Table 1). Because a high concentration of betaine aldehyde is toxic to plant cells (Rathinasabapathi et al. 1994), it is unlikely that LcBADH1 functions as a BADH in *L. chinensis*. In addition, the *V*_max_ value of LcBADH1 and LcBADH2 for betaine aldehyde was 1.59 and 1.26 U mg^−1^ protein, respectively. *V*_max_/*K*_m_, a criterion for catalytic activity, determined for betaine aldehyde of LcBADH1 was about 200-fold lower than that of LcBADH2. The kinetic characteristics of recombinant LcBADH1 and LcBADH2 proteins showed similar tendencies with that of the recombinant BBD1 and BBD2 proteins from barley, respectively (Table 2).

**Table 1:** **Substrate specificity of recombinant LcBADH1 and LcBADH2 proteins**

		**LcBADH1**			**LcBADH2**	
**Substrates**	***K***_**m**_ **(μM)**	***V***_**max**_ **(U mg** ^**-1**^ **protein)**	***V***_**max**_ **/** ***K***_**m**_	***K***_**m**_ **(μM)**	***V***_**max**_ **(U mg** ^**-1**^ **protein)**	***V***_**max**_ **/** ***K***_**m**_
Betaine aldehyde	27300 ± 7000	1.59 ± 0.16	5.96 × 10^−5^ ± 1.22 × 10^−5^	109 ± 10.0	1.26 ± 0.05	1.16 × 10^−2^ ± 1.55 × 10^−4^
AB-ald	48.1 ± 12.6	1.29 ± 0.08	3.55 × 10^−2^ ± 1.28 × 10^−2^	1.96 ± 0.30	1.01 ± 0.04	5.17 × 10^−1^ ± 3.27 × 10^−2^
AP-ald	27.4 ± 6.30	2.16 ± 0.14	1.03 × 10^−1^ ± 3.10 × 10^−2^	4.53 ± 0.81	2.70 ± 0.17	5.96 × 10^−1^ ± 2.85 × 10^−2^
TMAB-ald	56.5 ± 17.0	1.33 ± 0.19	2.91 × 10^−2^ ± 5.69 × 10^−3^	22.1 ± 1.50	1.10 ± 0.02	5.03 × 10^−2^ ± 4.18 × 10^−3^
TMAP-ald	762 ± 175	1.01 ± 0.08	1.22 × 10^−3^ ± 1.95 × 10^−4^	91.4 ± 9.60	1.81 ± 0.07	2.00 × 10^−2^ ± 4.14 × 10^−4^
NAD^+^	15.6 ± 5.70	1.36 ± 0.12	1.11 × 10^−1^ ± 3.69 × 10^−2^	7.66 ± 0.60	0.683 ± 0.01	9.02 × 10^−2^ ± 9.34 × 10^−3^
NADP^+^		N.D.		3680 ± 725	0.273 ± 0.02	7.60 × 10^−5^ ± 8.29 × 10^−6^

For kinetic analyses of the substrates, reaction mixtures contained 50 mM HEPES-KOH (pH 8.0), 500 μM NAD^+^ and various concentrations of each substrates. For kinetic analyses of NAD^+^ and NADP^+^, reaction mixtures contained 50 mM HEPES-KOH (pH 8.0), betaine aldehyde (5 mM for LcBADH1; 200 μM for LcBADH2) and the various concentrations of NAD^+^ or NADP^+^. Enzyme activities were determined as described in Materials and Methods. Values represent the mean of three experiments ± SE. AB-ald, 4-aminobutyraldehyde; AP-ald, 3-aminopropionaldehyde; TMAB-ald, 4-*N*-trimethylaminobutyraldehyde; TMAP-ald, 3-*N*-trimethylaminopropionaldehyde. N.D.; not detected.

**Table 2:** **Comparison of the specificity of** ***Leymus chinensis*** **and barley BADH proteins against betaine aldehyde**

**Protein**	***K***_**m**_ **(μM)**	***V***_**max**_ **(U mg** ^**-1**^ **protein)**	***V***_**max**_ **/** ***K***_**m**_	**Reference**
LcBADH1	27300 ± 7000	1.59 ± 0.16	5.96 × 10^−5^ ± 1.22 × 10^−5^	
				This study
LcBADH2	109 ± 10.0	1.26 ± 0.05	1.16 × 10^−2^ ± 1.55 × 10^−4^	
BBD1	19900 ± 3900	1.07 ± 0.10	5.37 × 10^−5^	Fujiwara et al. (2008)
BBD2	18.9 ± 1.40	2.05 ± 0.06	1.08 × 10^−1^	

Kinetic analyses were performed as described in Table 1. Values represent the mean of three experiments ± SE. The values for Barley BBD1 and BBD2 were cited from Fujiwara et al. (2008).

With regards to NAD^+^, the apparent *K*_m_ value of LcBADH1 and LcBADH2 was 15.6 μM and 7.66 μM, respectively (Table 1). Also, when NADP^+^ was used as an electron acceptor, the apparent *K*_m_ value of LcBADH2 was 3680 μM, which indicates that LcBADH2 prefers NAD^+^ to NADP^+^ as an electron acceptor (Table 1). However, LcBADH1 showed little BADH activity when NADP^+^ was used as an electron acceptor.

### ω-aminoaldehyde and *N*-trimethylaminoaldehyde dehydrogenase activities of LcBADH1 and LcBADH2

As shown in Additional file 2, both LcBADH1 and LcBADH2 catalyzed the dehydrogenation of 4-aminobutyraldehyde (AB-ald), 3-aminopropionaldehyde (AP-ald), 4-*N*-trimethylaminobutyraldehyde (TMAB-ald) and 3-*N*-trimethylaminopropionaldehyde (TMAP-ald). The *K*_m_ values of LcBADH1 for AB-ald, AP-ald, TMAB-ald and TMAP-ald were 48.1 μM, 27.4 μM, 56.5 μM and 762 μM, respectively (Table 1). With regards to LcBADH2, *K*_m_ values for AB-ald, AP-ald, TMAB-ald and TMAP-ald were 1.96 μM, 4.53 μM, 22.1 μM and 91.4 μM, respectively (Table 1). *V*_max_/*K*_m_ of LcBADH2 for AB-ald, AP-ald, TMAB-ald and TMAP-ald was higher than that of LcBADH1, indicating that LcBADH2 exhibited relatively high affinity for the ω-aminoaldehyde and *N*-trimethylaminoaldehyde tested in this study compared with LcBADH1.

### Effect of NaCl treatment on the BADH activity of recombinant LcBADH1 and LcBADH2

**Figure 3:**
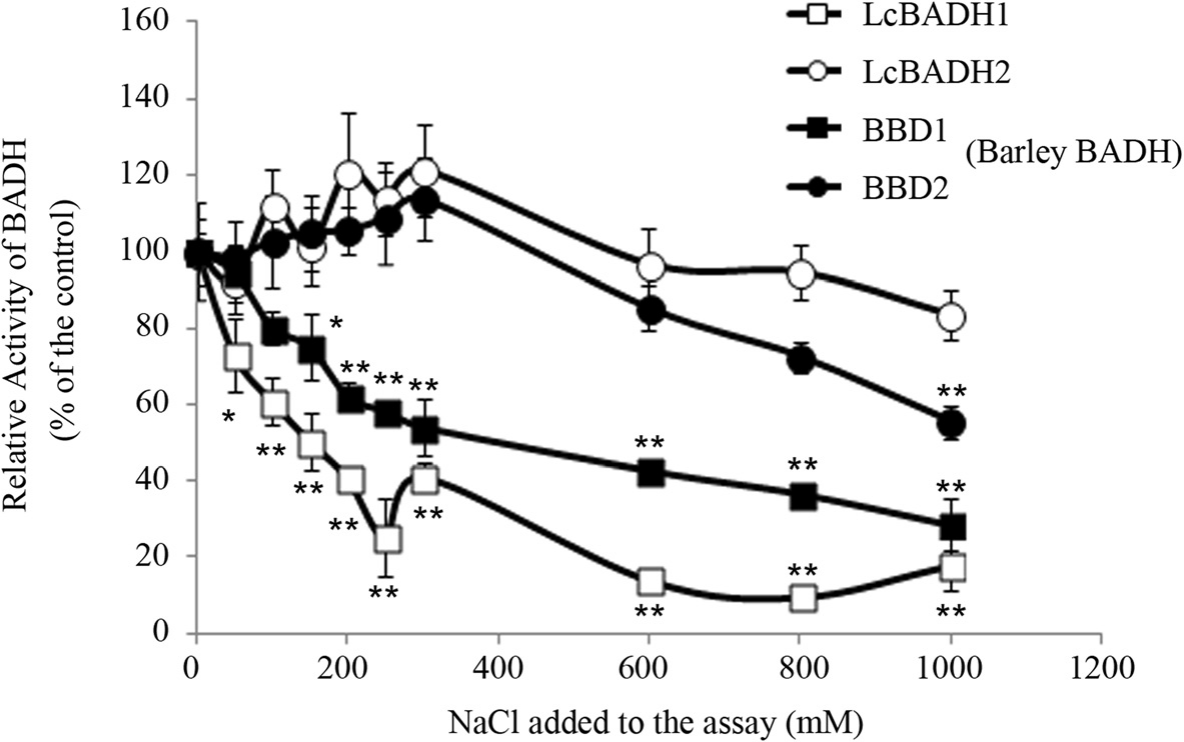
Effect of the application of NaCl on the activity of BADH proteins from *Leymus chinensis* and barley. Open squares and circles represent the recombinant protein of LcBADH1 and LcBADH2, respectively. The recombinant barley BADH proteins, BBD1 (closed square) and BBD2 (closed circle), were also used for the assay. The recombinant protein was incubated in the standard mixture supplemented with the indicated concentrations of NaCl for 5 min and used for the spectrophotometrical measurement of BADH activity as described in Materials and methods. Data represent the mean of three experiments ± SE (n = 3). Vertical bars represent SE. The symbols * and ** represent significant differences from control values in each protein at P < 0.05 and P < 0.01, respectively (Dunnett’s test).

We examined the stability of recombinant LcBADH1 and LcBADH2 proteins in the presence of NaCl. The recombinant barley BADH proteins, BBD1 and BBD2 (Fujiwara et al. 2008), were also used. After the recombinant proteins were incubated at the indicated concentrations of NaCl for 5 min, the dehydrogenation activity of betaine aldehyde was measured by adding 5 mM betaine aldehyde. The activity of LcBADH1 and BBD1 was significantly decreased by treatment with concentrations of NaCl greater than 50 mM and 150 mM, respectively (Figure 3). On the other hand, the activity of LcBADH2 and BBD2 proteins decreased only at NaCl concentrations greater than 300 mM (Figure 3). The activity of BBD2 protein decreased at concentrations of NaCl greater than 600 mM and significantly in the presence of 1 M NaCl. However, LcBADH2 protein showed comparable activity with the control at concentrations of NaCl up to 1 M.

### Tissue and subcellular localization of BADH and CMO-like proteins in *L. chinensis* leaves

**Figure 4:**
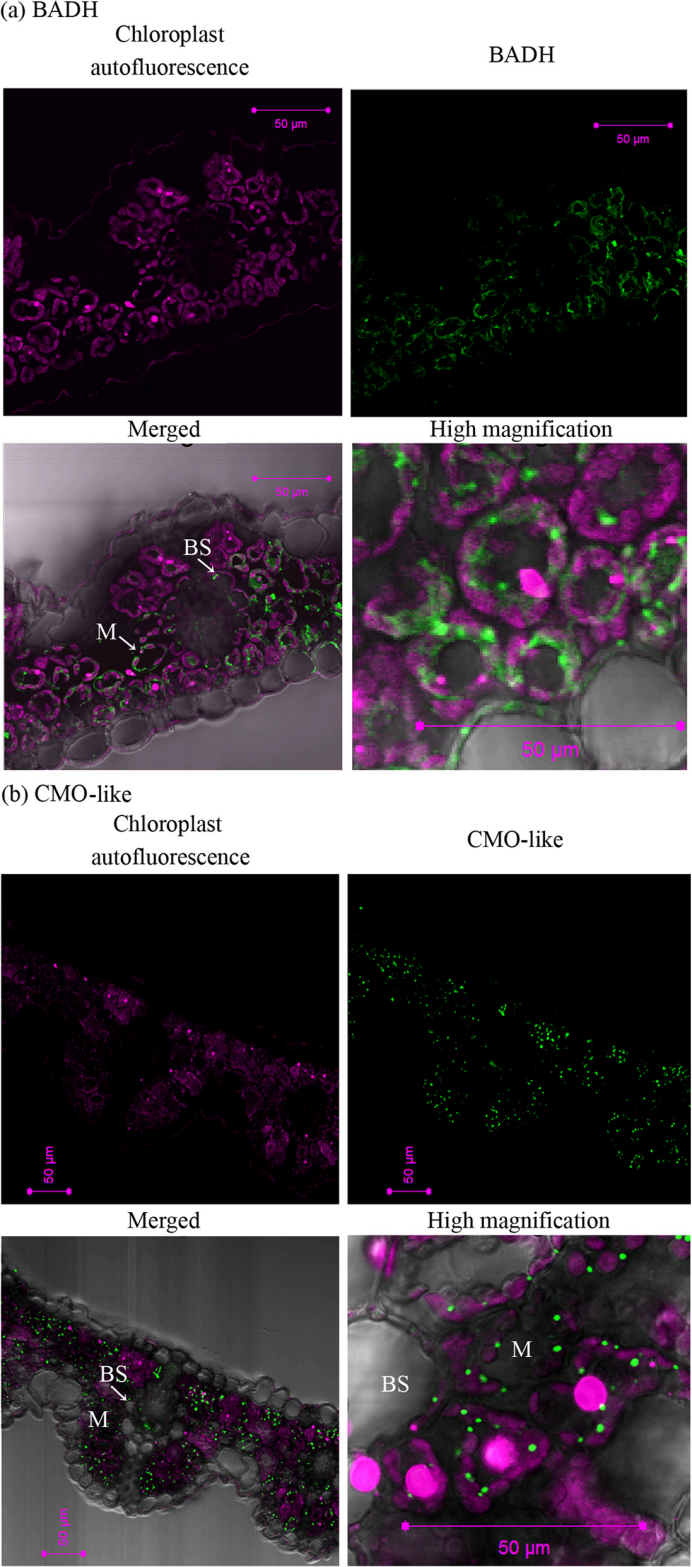
Subcellular localization of **(a)** BADH and **(b)** CMO-like proteins in the leaf blades of *Leymus chinensis* plants. The middle part of leaf blades of the youngest fully-expanded leaf of four-week-old *L. chinensis* plants was used. The magenta autofluorescence emitted by chloroplasts, the green fluorescence of BADH and CMO-like proteins detected with fluorescein-conjugated goat anti-rabbit antibody, merged image and enlarged image are shown. Scale bars represent 50 μm. *M* mesophyll, *BS* bundle sheath.

Leaf blades of *L. chinensis* plants grown under normal conditions were used to determine the tissue and subcellular localization of BADH proteins. The strong green fluorescent signals for BADH were detected in the cytosol and dot-shaped organelles in mesophyll and bundle sheath cells, which did not overlap with the autofluorescence of chloroplasts (shown as magenta) (Figure 4a).

We also examined the localization of CMO-like proteins in the leaves using the anti-HvCMO peptide antibody (Mitsuya et al. 2011). The fluorescent signals for CMO-like proteins were detected in the non-chloroplastic dot-shaped organelles in mesophyll and bundle sheath cells (Figure 4b), which is similar to barley where CMO is localized in peroxisomes in mesophyll and bundle sheath cells (Mitsuya et al. 2011).

We also used the leaf blades grown under saline conditions but the localization of BADH and CMO proteins was comparable to those under normal conditions (data not shown).

### Effect of NaCl on the accumulation of GB, choline and GB-biosynthetic proteins in *L. chinensis* and barley

**Figure 5:**
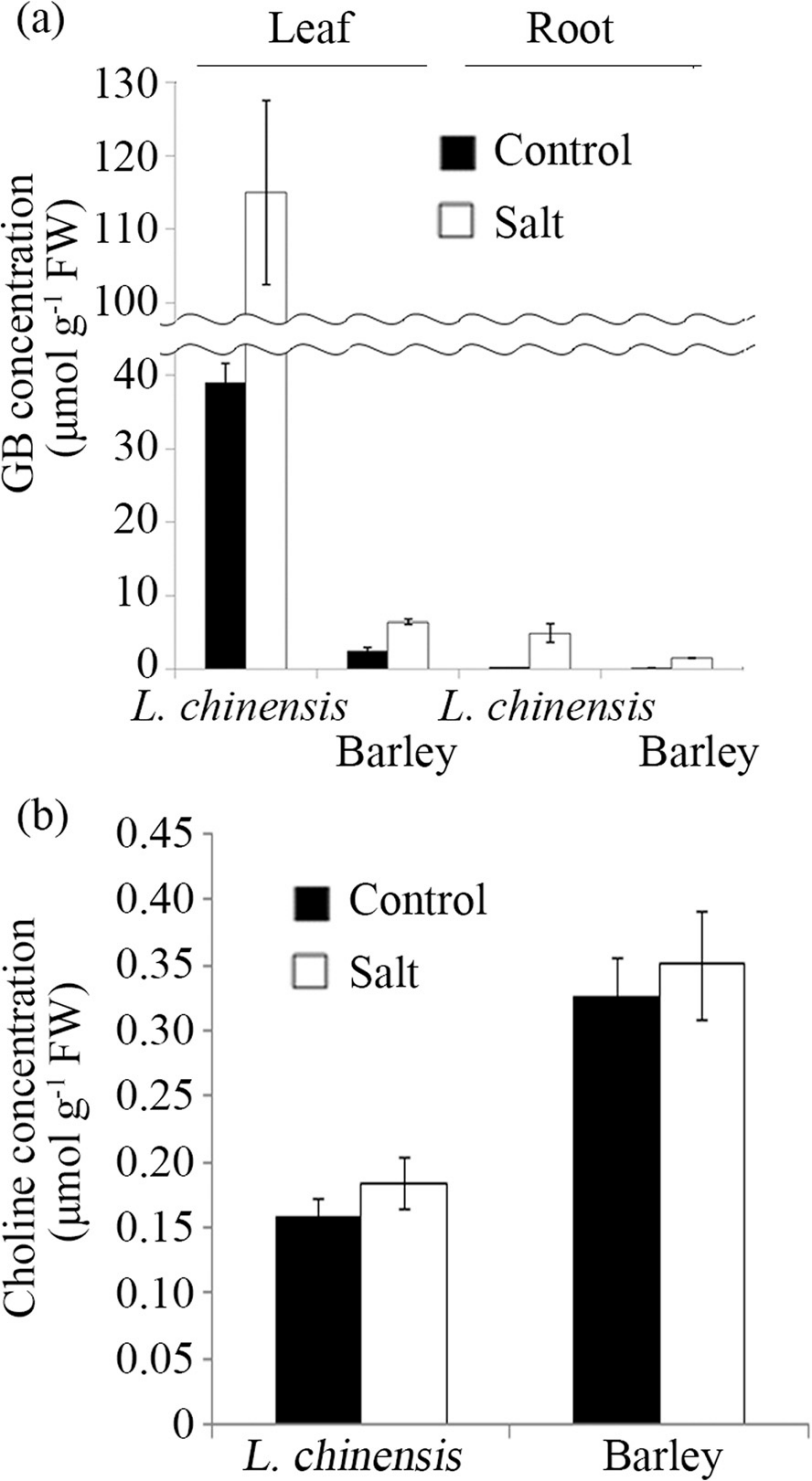
Effect of the application of NaCl on the accumulation of GB and choline in *Leymus chinensis* and barley plants. Four-week-old *L. chinensis* and three-week-old barley plants grown hydroponically with same leaf stage (the youngest fully-expanded leaf was sixth from the bottom) were treated with 0 and 300 mM NaCl for 72 h. The sixth leaf blades and roots were harvested and used for the analyses. **(a)** The concentration of GB in the sixth leaf blades and roots. **(b)** The concentration of choline in the sixth leaf blades. Data are means ± SE (n = 3). Vertical bars represent SE.

The concentration of GB in the sixth leaf blades and roots of *L. chinensis* and barley under normal and saline conditions were measured. Because *L. chinensis* grows slower than barley, *L. chinensis* and barley plants were hydroponically grown for four and three weeks respectively, to obtain plants with the same leaf stage (the youngest fully-expanded leaf was sixth from the bottom). Four-week-old *L. chinensis* and three-week-old barley plants were then treated with 300 mM NaCl for 72 h. The concentration of GB increased significantly in the presence of NaCl in the sixth leaf blades and roots of both plants and was higher in the leaves than in the roots (Figure 5a). *L. chinensis* accumulated much more GB than barley under both normal and saline conditions (Figure 5a).

The concentration of choline, a precursor of GB, was also measured using sixth leaf blades of *L. chinensis* and barley plants. Barley showed higher concentrations of choline than *L. chinensis*, although the concentration was not significantly affected by NaCl treatment in both plants (Figure 5b).

**Figure 6:**
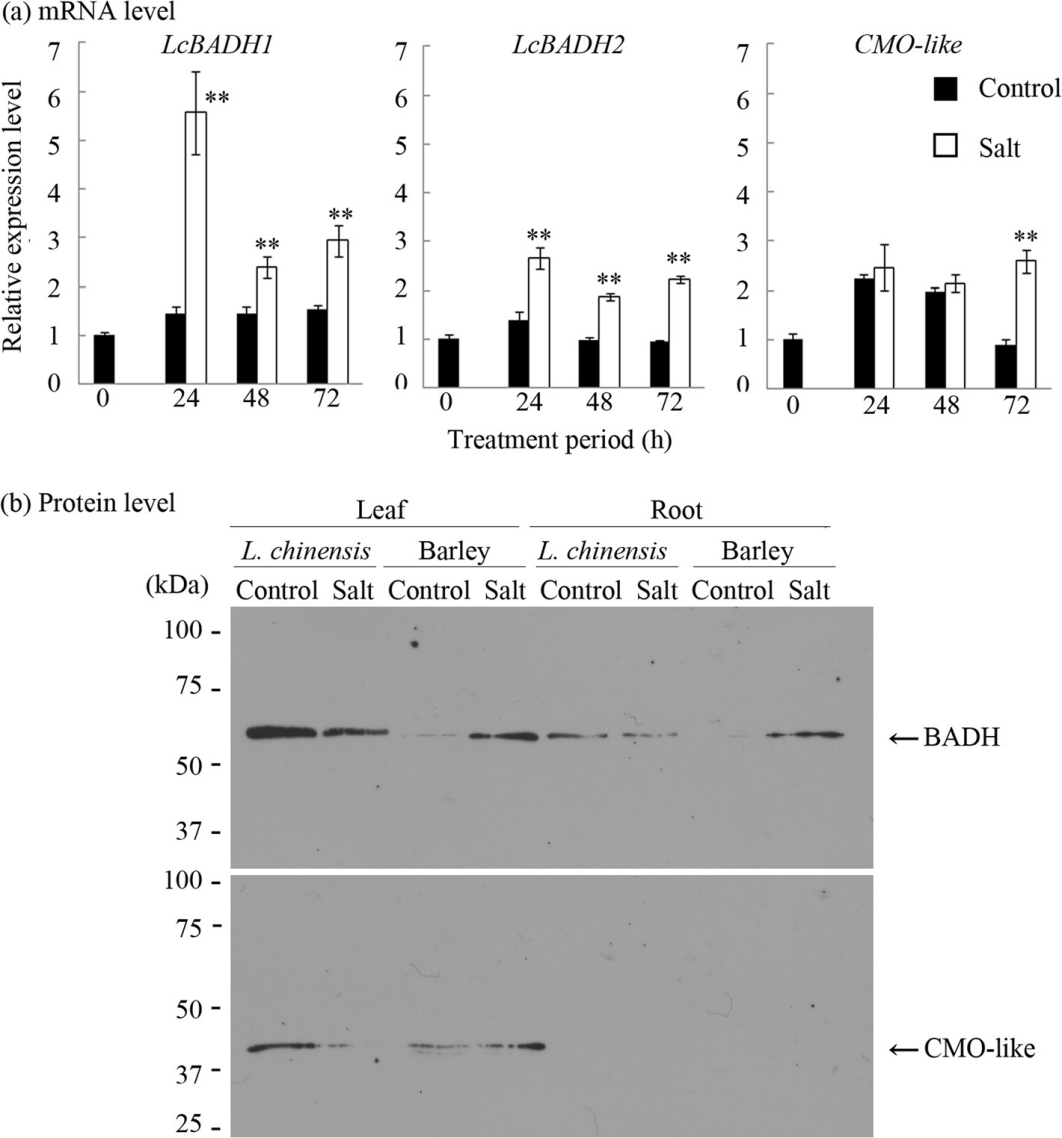
Expression pattern of mRNA and proteins of BADH and CMO-like in *Leymus chinensis* and barley plants under normal and saline conditions. Four-week-old *L. chinensis* and three-week-old barley plants grown hydroponically with same leaf stage (the youngest fully-expanded leaf was sixth from the bottom) were treated with 0 and 300 mM NaCl for 72 h. The sixth leaf blades and roots were harvested and used for the analyses. **(a)** Real-time PCR analysis of the transcript level of *LcBADH1*, *LcBADH2* and *CMO*-like genes under normal and saline conditions in the sixth leaf blades. *LcTubulin* was used as an internal control to normalize for variation in the amount of cDNA template. Data show the mean of relative values with one unit being control (0 h) ± SD of three biological replicates. The symbols ** represent significant differences from control values at each treatment period at P < 0.01 (Student’s *t*-test). **(b)** Protein gel-blot analyses of BADH and CMO-like proteins. The lanes were loaded with protein extracted from sixth leaf blades and roots equivalent to 0.25 mg FW. The sizes of molecular mass standards are shown on the left in kDa.

The level of transcripts of *LcBADH1* and *LcBADH2* genes in the sixth leaf blades of *L. chinensis* was examined using real-time PCR. The levels of *LcBADH1* and *LcBADH2* mRNAs increased in the presence of 300 mM NaCl and showed peak accumulation at 24 h (Figure 6a). The *CMO*-like mRNA level under saline conditions, determined using the EST information (Genbank accession number EU003877), was comparable with that under control conditions up to 48 h, but had increased at 72 h (Figure 6a).

We also determined the protein level of BADH and CMO-like in the sixth leaf blades and roots of *L. chinensis* and barley plants treated with 0 and 300 mM NaCl for 72 h. The protein level of BADH and CMO-like was greater in the leaves of both plants than in the roots (Figure 6b). The amount of BADH and CMO-like proteins was greater in *L. chinensis* than in barley under normal conditions. On the other hand, the expression level of BADH protein was increased in barley but not in *L. chinensis* in the presence of NaCl. We also determined BADH activity using crude protein extracted from the sixth leaf blades of *L. chinensis* plants. The BADH activity in *L. chinensis* leaves under normal and saline conditions was 0.51 and 0.54 nmol min^−1^ mg^−1^ protein, respectively, which was much higher than the activity in barley leaves (0.03 and 0.11 nmol min^−1^ mg^−1^ protein under normal and saline conditions, respectively) (Ishitani et al. 1993) (Table 3).

**Table 3:** **BADH activity in the leaves of** ***Leymus chinensis*** **and barley plants grown under normal and salinized conditions**

**Species**	**Treatment**	**BADH activity (nmol min** ^**-1**^ **mg** ^**-1**^ **protein)**	**Reference**
*L. chinensis*	Control	0.51 ± 0.023	This study
	NaCl	0.54 ± 0.048	
Barley	Control	0.03	Ishitani et al. (1993)
	NaCl	0.11	

## Discussion

### The GB-biosynthetic BADH protein in *L. chinensis*

We have isolated cDNAs for two kinds of *BADH* genes, *LcBADH1* and *LcBADH2* from *L. chinensis* and have shown that LcBADH1 and LcBADH2 proteins were putatively localized to peroxisome-like dot-shaped organelles and cytosol, respectively, in mesophyll and bundle sheath cells in the leaves (Figure 4). It is suggested that cytosolic LcBADH2 showing high affinity to betaine aldehyde (true BADH; Fitzgerald et al. 2009) plays a major role in GB biosynthesis in *L. chinensis* whereas LcBADH1 shows much low affinity to betaine aldehyde (highly BADH homology aminoaldehyde dehydrogenase; Fitzgerald et al. 2009). The result was consistent with other graminaceous plants such as barley and rice that have a true BADH (BBD2 in barley and OsBADH2 in rice) and a highly BADH homology aminoaldehyde dehydrogenase (BBD1 in barley and OsBADH1 in rice) (Fujiwara et al. 2008; Mitsuya et al. 2009). Díaz-Sánchez et al. (2012) has reported that, in spinach BADH, small amino acid residues such as alanine or cysteine at position 441 is critical for the high affinity to betaine aldehyde whereas isozymes possessing isoleucine at the position show low affinity to betaine aldehyde. LcBADH2 and BBD2 with high affinity to betaine aldehyde also possess cysteine and LcBADH1, BBD1, and OsBADH1 with low affinity to betaine aldehyde possess isoleucine at the corresponding position, which corresponds to the result of Díaz-Sánchez et al. (2012), however OsBADH2 has a isoleucine at this position but showed moderately high affinity to betaine aldehyde (Mitsuya et al. 2009).

Together with the result of subcelullar localization of CMO-like protein (Figure 4), it is indicated that *L. chinensis* produces GB by oxidizing choline in a co-operative way via dot-shaped organelles and cytosol in mesophyll and bundle sheath cells in the leaves. It is similar to GB production that is performed in two separated subcellular compartments (peroxisomes and cytosol) in barley (Mitsuya et al. 2011, Mitsuya et al. 2013). This result supports that graminaceous plants have a non-chloroplastic pathway of GB production unlike Amaranthaceae (Weigel et al. 1988).

### The comparison of the characteristics of BADH proteins between *L. chinensis* and barley

Next, we compared the characteristics of the GB-biosynthetic BADH proteins between *L. chinensis* and barley showing different levels of GB accumulation (Figure 5a). The catalytic efficiency of BADH and its subcellular and tissue localization were comparable between *L. chinensis* and barley (Table 2 and Figure 4), which indicates that those characteristics were not correlated with the significant difference of GB accumulation between two graminaceous plants. In addition, the concentration of choline, a precursor of GB, was rather higher in barley than in *L. chinensis* (Figure 5b). On the other hand, the expression analysis of BADH and CMO proteins indicates that much larger GB accumulation in *L. chinensis* in comparison to barley can be correlated with constant higher expression level of BADH (and CMO-like) proteins under normal conditions in *L. chinensis* (Table 3, Figure 6). Because GB is a stable end product of metabolism (Grattan and Grieve 1985), it is indicated that constitutive production of GB at high levels under normal conditions during the growth causes high accumulation of GB in *L. chinensis*.

### The mechanism of salinity-induced increase of GB production in *L. chinensis*

The accumulation of GB was enhanced by salinity in *L. chinensis* (Figure 5a), but the expression level of BADH and CMO-like proteins did not increase in the presence of NaCl (Figure 6). Also, the size of the free choline pool in the leaves of *L. chinensis* was comparable between normal and salinized conditions (Figure 5b). At the moment, it is difficult to conclude how salinity induced the increased production of GB in *L. chinensis*. So far, it was reported that the increase of GB accumulation in the presence of NaCl is caused by the increased expression and activity of BADH or choline oxidizing protein in graminaceous plants (Ishitani et al. 1993, Nakamura et al. 2001, Su et al. 2006). From the data in this study, it is indicated that the expression of BADH protein is not a limiting factor for GB synthesis under salinity in *L. chinensis*. It is possible to suggest that the metabolic flux to choline from its precursors [ethanolamine and successively-methylated ethanolamine derivatives catalyzed by phosphoethanolamine *N*-methyltransferase (PEAMT)] may be increased and the increased choline is immediately converted to betaine aldehyde and GB in salinized *L. chinensis*. In transgenic tobacco expressing spinach CMO, the supply of endogenous choline or its precursors mono-and di-methylethanolamine is one of the limiting factors of GB biosynthesis (Nuccio et al. 1998). Moreover, the overexpression of PEAMT enhances the production of choline and GB in tobacco (McNeil et al. 2000). The regulation of PEAMT could be the next target to determine the mechanism of how GB production was increased by salinity in *L. chinensis*.

Since GB is produced in a co-operative way in the cytosol and dot-shaped organelles in *L. chinensis* (Figure 4), it is also necessary to determine whether the increase of GB under salinity is attributed by the increased activity of the transport of choline and/or betaine aldehyde in *L. chinensis*. It was previously shown that, in tobacco plants that express spinach CMO in the chloroplasts, the import of choline into the chloroplasts is a major constraint on GB synthesis (McNeil et al. 2000).

### Effect of NaCl treatment on the BADH activity of recombinant LcBADH1 and LcBADH2

Interestingly, recombinant proteins of LcBADH2 and barley BBD2 showed no reduction of its activity under the treatment of NaCl up to 300 mM (Figure 3). This result indicates that those proteins can function in the presence of reasonable concentrations of NaCl in the intracellular space. In contrast, it was reported that the recombinant BADH proteins from spinach, salt-tolerant mangrove *Avicennia marina* and *E. coli* showed 30 to 60% inhibition in the presence of 300 mM NaCl *in vitro* (Hibino et al. 2001; Oishi and Ebina 2005). These indicate that LcBADH2 and BBD2 are more tolerant to NaCl in comparison to other plants’ BADH. By identifying the protein domains that allow LcBADH2 and BBD2 to maintain the activity under high concentrations of NaCl, it could be possible to improve the salt tolerance of other plants’ BADH.

### Other possible functions of LcBADH1 and LcBADH2 proteins

As well as the dehydrogenation of betaine aldehyde, LcBADH1 and LcBADH2 catalyzed the dehydrogenation of ω-aminoaldehyde (AB-ald and AP-ald) and *N*-trimethylaminoaldehyde (TMAB-ald and TMAP-ald) (Table 1). It was reported that not only BADHs from barley, rice, spinach and *Zoysia tenuifolia*, but also human and *E. coli* BADHs oxidize AB-ald and AP-ald (Chern and Pietruszko 1995; Trossat et al. 1998; Incharoensakdi et al. 2000; Livingstone et al. 2003; Oishi and Ebina 2005; Bradbury et al. 2008; Fujiwara et al. 2008). The affinity to aminoaldehydes was not so different between LcBADH1 and LcBADH2 or among LcBADHs and other plants’ BADHs unlike that to betaine aldehyde. In addition, LcBADH1 and LcBADH2 showed higher affinity to aminoaldehydes in comparison to that of betaine aldehyde, which is consistent with other plants’ BADH (Livingstone et al. 2003; Oishi and Ebina 2005; Fujiwara et al. 2008; Mitsuya et al. 2009). AB-ald and AP-ald are known as intermediates in polyamine degradation in plants, although physiological functions of AB-ald and AP-ald are still unclear (Awal et al. 1995; Binda et al. 1998). It was reported that rice BADH protein (OsBADH2) has a role in the dehydrogenation of AB-ald to produce γ-aminobutyric acid (GABA) (Chen et al. 2008). GABA is also accumulated in response to various abiotic stresses such as salinity (Kinnersley and Turano 2000). Moreover, impaired GABA transaminase which functions in GABA catabolism, causes salt hypersensitivity in *Arabidopsis* plants (Renault et al. 2010). Therefore, LcBADH1 and LcBADH2 may possibly contribute to salt tolerance via the biosynthesis of GABA as well as GB in *L. chinensis*.

TMAB-ald and TMAP-ald are derived from AB-ald and AP-ald by the trimethylation of primary amino group and also catalyzed by barley BBD1 and BBD2 (Fujiwara et al. 2008). TMAB-ald is the intermediate of carnitine synthesis in mammals and some microorganisms (Vaz and Wanders 2002; Hassan et al. 2007). Since information on the physiological function of TMAP-ald in plants is unavailable, further study is needed to elucidate the physiological role of LcBADH proteins in *L. chinensis* plants.

## Conclusions

We have isolated cDNAs for *LcBADH1* and *LcBADH2* xgenes which encode two kinds of BADH protein in *L. chinensis* plants, a GB-hyperaccumulating graminaceous halophyte. The analysis of enzyme kinetics indicated that cytosolic LcBADH2 protein plays a major role in GB biosynthesis. In addition, the recombinant LcBAEDH2 protein was tolerant to NaCl whereas LcBADH1 wasn’t. It is indicated that *L. chinensis* produces GB by oxidizing choline in a co-operative way via dot-shaped organelles and cytosol in mesophyll and bundle sheath cells in the leaves. The kinetics, subcellular and tissue localization of BADH proteins were comparable between *L. chinensis* and barley, a Graminaceae accumulating less amounts of GB. The activity and expression level of BADH proteins were higher in *L. chinensis* compared with barley under both normal and salinized conditions, which may be related to the significant difference in the amount of GB accumulation between two plants.

## Methods

### Plant materials and growth conditions

*Leymus chinensis* (Trin.) Tzvel. plants consist of two ecotypes, and, the gray type of *L. chinensis* was grown in soil in a greenhouse of Nagoya University (Nagoya, Japan, 35°9’N, 136°58’E). The illumination and temperature were not controlled. The plant is perennial and developed stolons and shoots from the nodes during April to October. Therefore, the experiments using plants were conducted during this period of the year.

For NaCl stress treatment, the developed stolons containing some nodes were cut, put into vermiculite and grown hydroponically in the culture solution [2.5 mM KH_2_PO_4_, 1 mM MgSO_4_, 5 mM KNO_3_, 1 mM Ca(NO_3_)_2_, 0.1 mM Fe-EDTA, 70 μM H_3_BO_3_, 1 μM MnCl_2_, 0.5 μM CuSO_4_, 1.54 μM (NH_4_)_6_Mo_7_O_24,_ pH 5.5], where axillary buds were grown into plantlets. Plants were grown in a growth chamber with 13 h of illumination (about 200 μmol m^−2^ s^−1^ at plant level, 25°C) and 11 h of darkness at 20°C. After the emergence, the plantlets were grown for four weeks, and treated with salinity stress. For NaCl treatment, 4-week-old plants were treated with the nutrient solution containing 300 mM NaCl and grown for further 72 h. Salinity treatment was initiated one hour after the start of the light period.

We have also used barley (*Hordeum vulgare* L. cv. Haruna-nijyo) for the comparison with *L. chinensis*. Seeds of barley were surface sterilized and grown hydroponically as described previously (Fujiwara et al. 2010). Three-week-old seedlings were treated with the nutrient solution containing 300 mM NaCl and grown for further 72 h.

After treatment, plants were collected at the indicated period and used for further experiments. After treatment, plants were collected at the indicated period, immediately frozen with liquid N_2_ and preserved at −80°C until use. For immunofluorescent labeling experiments, fresh plant samples were used for excising the segments and fixiation using a fixation buffer, according to Mitsuya et al. (2011).

### Cloning of *LcBADH* cDNAs and construction of expression vectors

The fragment of *LcBADH* cDNA was amplified by PCR from cDNA of the leaves of *L. chinensis* using degenerate primers (BADHF and BADHR; Additional file 3) designed on the basis of the highly conserved amino acid sequences from several plant BADH cDNAs of *Atriplex hortensis* (Xiao et al. 1995), sugar beet (McCue and Hanson 1992), spinach (Weretilnyk and Hanson 1990), barley (Ishitani et al. 1995) and rice (Nakamura et al. 1997). A cDNA library of the leaves of salt-treated *L. chinensis* plants was constructed as described previously (Inada et al. 2005). A partial cDNA fragment of the *LcBADH* gene which was obtained by the above PCR was used as a probe to screen the cDNA library. The ^32^P-labeled probe was prepared using the Megaprime DNA labeling system (Amersham Bioscience, Uppsala, Sweden). After library screening, the positive candidates were excised with pBluescript SK(−) vector, and sequenced as described previously (Inada et al. 2005). Because the 5’-terminus of the open reading frame (ORF) of *LcBADH1* gene was not included in the clone, the cDNA sequence containing the entire coding region was obtained by 5’-RACE. THe 5’-RACE-PCR was performed with the isolated total RNAs of *L. chinensis* by a SMART RACE cDNA amplification kit (Clontech, Palo Alto, CA, USA). Sequence data from this article has been deposited at DDBJ/EMBL/GenBank under accession number AB711137 (for *LcBADH1*) and AB183716 (for *LcBADH2*). The primers used in this study are listed in Additional file 3.

### Sequence alignment and construction of a phylogenetic tree

The deduced amino acid sequences of *LcBADH1* and *LcBADH2* were aligned by the N-J method using ClustalX software (Larkin et al. 2007). The phylogenetic tree was built using the MEGA6 program (Tamura et al. 2013). The tree was inferred by using the maximum likelihood method with 1000 bootstrap replicates. The initial tree for the heuristic search was obtained automatically by applying Neibor-Join and BioNJ algorithms to a matrix of pairwise distances estimated using a JTT model, and then selecting the topology with a superior log likelihood value. The tree was drawn to scale, with branch lengths measured in the number of substitutions per site. All positions with less than 50% site coverage were eliminated.

### Purification of recombinant BADH proteins

The ORF of *LcBADH1* and *LcBADH2* was amplified by PCR using the primer set of LcB1sen3 and LcB1ant3, LcB2sen3 and LcB2ant3, respectively (Additional file 3). They were cloned into pET32a vector (Novagen, Madison, WI). Recombinant LcBADH1 and LcBADH2 proteins were obtained as described previously (Fujiwara et al. 2008). The recombinant LcBADH1 and LcBADH2 proteins were subjected to SDS-PAGE to confirm their homogeneous purification. Recombinant barley BBD1 and BBD2 proteins were also obtained as described previously (Fujiwara et al. 2008). Protein concentration was determined using the Bio-Rad Bradford Protein Assay (Bio-Rad, Hercules, CA, USA) using BSA as a standard.

### Enzyme assays

Betaine aldehyde chloride was obtained from Sigma-Aldrich (St Louis, MO). The diethylacetals of AB-ald and AP-ald were obtained from Sigma-Aldrich and Tokyo Kasei Kogyo (Tokyo), respectively. TMAB-ald and TMAP-ald were distributed by Professor Nobuhiro Mori (Tottori University, Japan). They were hydrolyzed as described previously (Fujiwara et al. 2008) and neutralized by adding equivalent volume of KOH. Aldehyde dehydrogenase activities were determined as described previously (Fujiwara et al. 2008). One unit of enzyme activity is defined as the amount of enzyme that catalyzes the formation of 1 μmol of NADH per min.

The effect of NaCl on the recombinant BADHs was determined by incubating purified enzymes (2 μg) in 50 mM HEPES buffer (pH 8.0), 0.5 mM NAD^+^ and the indicated concentrations of NaCl for 5 min at 30°C. After pre-incubation, 5 mM betaine aldehyde was added and the activity was measured.

### Real-time PCR

RNA isolation and real-time PCR were done as described previously (Mitsuya et al. 2005, Mitsuya et al. 2009). The primers used in this study are shown in Additional file 3. *LcTubulin* (accession number CN466136) was used as the internal control. The transcript level of target genes was normalized to that of *LcTubulin* (value = 1).

### Assays of BADH activity in *L. chinensis* leaves

The extraction of total soluble protein from the sixth leaf blades of *L. chinensis* plants and BADH assay by the fluorometric method were done as described previously (Arakawa et al. 1990).

### Protein gel-blot analyses of BADH and CMO proteins

Total soluble protein was extracted from the sixth leaf blades of *L. chinensis* and barley plants as described previously (Burnet et al. 1995). For protein gel-blot analysis, proteins were electrophoresed on SDS-polyacrylamide gels, transferred to a polyvinylidene fluoride membrane and developed using an ECL Advance Western Blotting Detection Kit (GE healthcare biosciences, Piscataway, NJ, USA). Primary [rabbit anti-barley HvCMO peptides (Mitsuya et al. 2011) and anti-spinach BADH (Arakawa et al. 1992) IgGs] and secondary (peroxidase-conjugated donkey anti-rabbit IgG, GE Healthcare) antibodies were diluted in Can Get Signal solution (Toyobo, Osaka, Japan) at 1:1,000, 1:5,000 and 1:5,000, respectively. It was found that the anti-barley HvCMO peptides antibody can cross-react with CMO-like proteins in *L. chinensis* (Figures 3, 6). In addition, the partial sequence of CMO-like protein of *L. chinensis* (GenBank Accession No. ABV64740) is highly similar to that of barley HvCMO protein (93% similarity) and the sequence of the two antigen peptides for anti-HvCMO peptides antibody is conserved between the two plant species.

### Determination of the concentration of GB and choline

The concentration of GB was determined as reported previously (Jagendorf and Takabe 2001).

The extraction of choline from leaves was done according to Toyosawa and Nishimoto (1967). The choline was reacted with 1-naphthyl isocyanate to form a stable cationic aromatic urethane and measured by high performance liquid chromatography on a cation exchange column, followed by fluorescence detection as described in McEntyre et al. (2009).

### Immunofluorescent labeling of BADH and CMO proteins

Immunodetection of BADH and CMO proteins was performed as described previously (Mitsuya et al. 2011). Rabbit anti-spinach BADH (Arakawa et al. 1992) and Anti-HvCMO peptides (Mitsuya et al. 2011) IgGs were used for detection of BADH and CMO, respectively. Fluorescein isothiocyanate-conjugated goat anti-rabbit IgG (Wako Pure Chemical Industries, Osaka, Japan) was used as a secondary antibody. The sections were observed using a confocal microscope (Carl Zeiss LSM 5 PASCAL). At least 3 samples were used for the analysis.

## Additional files

Additional file 1:
**SDS-PAGE of recombinant LcBADH1 and LcBADH2.** LcBADH1 and LcBADH2 were expressed as fusion proteins in Escherichia coli and purified as described in Methods. After enterokinase treatment, 10 μg protein was electrophoresed. SDS-PAGE samples were stained using Coomassie brilliant blue R-250 and the size of molecular mass standards are shown on the left in kDa. Additional file 2:
**Kinetic analysis of betaine aldehyde,** **ω-aminoaldehyde** **and trimethylaminoaldehyde dehydrogenase activities of LcBADH1 and LcBADH2.** Assays were performed under the standad conditions as described in Methods. Substrate-dependent activities were plotted against substrate concentration. Data represent the mean of three experiments ± SE. AB-ald, 4-aminobutyraldehyde; AP-ald, 3-aminopropionaldehyde; TMAB-ald, 4-*N*-trimethylaminobutyraldehyde; TMAP-ald, 3-*N*-trimethylaminopropionaldehyde. Additional file 3:
**Primers used in this study.**

## Abbreviations

AB-ald: 4-aminobutyraldehyde
AP-ald: 3-aminopropionaldehyde
BADH: Betaine aldehyde dehydrogenase
CMO: Choline monooxygenase
GABA: γ-aminobutyric acid
GB: Glycinebetaine
ORF: Open reading frame
PEAMT: Phosphoethanolamine *N*-methyltransferase
TMAB-ald: 4-*N*-trimethylaminobutyrialdehyde
TMAP-ald: 3-*N*-trimethylaminopropionaldehyde
