# Supplementary material for: Functional and expression analyses of two kinds of betaine aldehyde dehydrogenases in a glycinebetaine-hyperaccumulating graminaceous halophyte, Leymus chinensis
Source: Springerplus. 2015 Apr 30;4:202. doi: 10.1186/s40064-015-0997-4 (PMC4431990; doi:10.1186/s40064-015-0997-4)
Supplement: Additional file 1: — SDS-PAGE of recombinant LcBADH1 and LcBADH2. LcBADH1 and LcBADH2 were expressed as fusion proteins in Escherichia coli and purified as described in Methods. After enterokinase treatment, 10 μg protein was electrophoresed. SDS-PAGE samples were stained using Coomassie brilliant blue R-250 and the size of molecular mass standards are shown on the left in kDa. [file 40064_2015_997_MOESM1_ESM.ppt]

## Slide 1
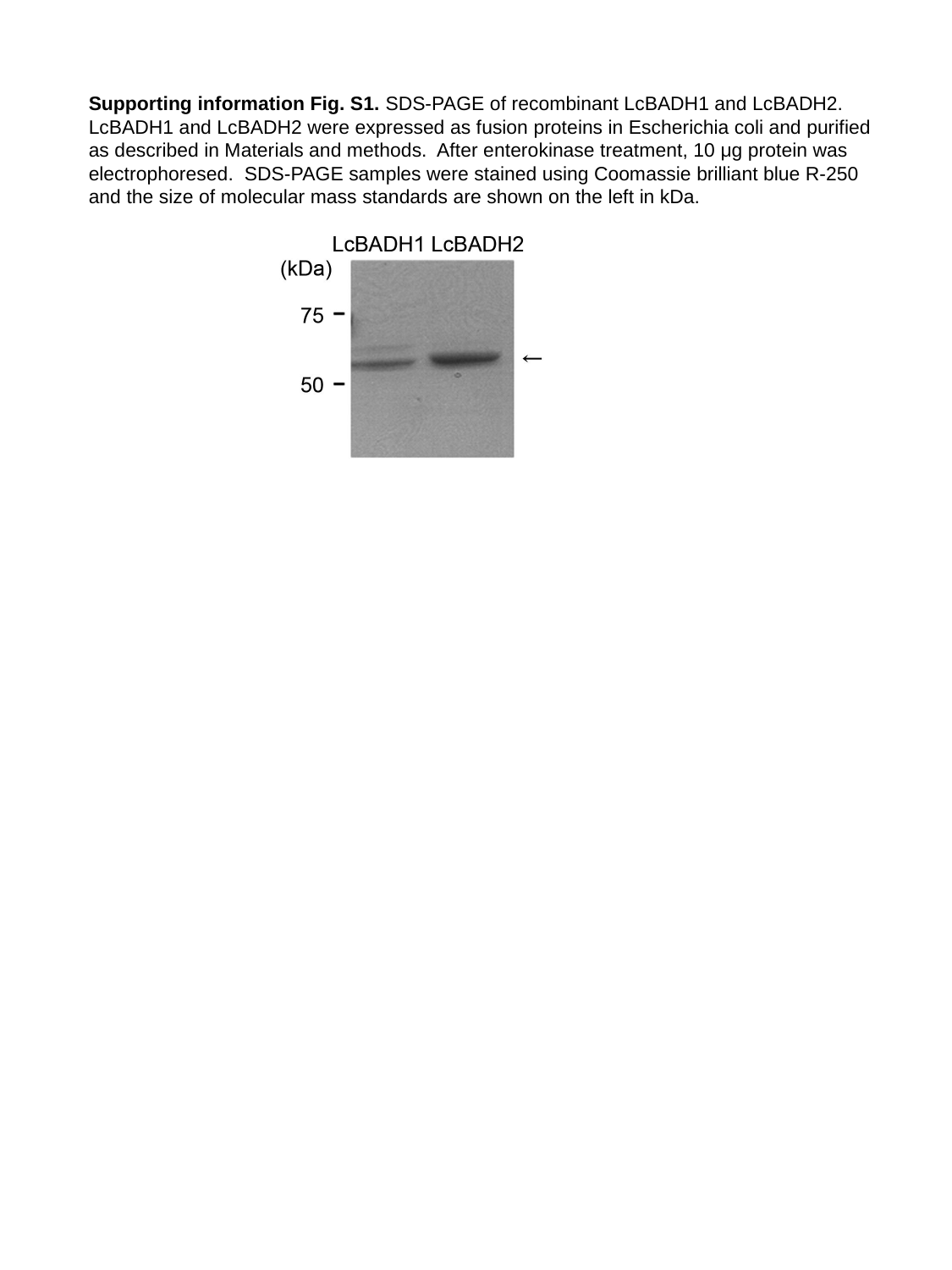

Supporting information Fig. S1. SDS-PAGE of recombinant LcBADH1 and LcBADH2. LcBADH1 and LcBADH2 were expressed as fusion proteins in Escherichia coli and purified as described in Materials and methods. After enterokinase treatment, 10 μg protein was electrophoresed. SDS-PAGE samples were stained using Coomassie brilliant blue R-250 and the size of molecular mass standards are shown on the left in kDa.
