# Supplementary material for: Functional and expression analyses of two kinds of betaine aldehyde dehydrogenases in a glycinebetaine-hyperaccumulating graminaceous halophyte, Leymus chinensis
Source: Springerplus. 2015 Apr 30;4:202. doi: 10.1186/s40064-015-0997-4 (PMC4431990; doi:10.1186/s40064-015-0997-4)
Supplement: Additional file 2: — Kinetic analysis of betaine aldehyde, ω-aminoaldehyde and trimethylaminoaldehyde dehydrogenase activities of LcBADH1 and LcBADH2. Assays were performed under the standad conditions as described in Methods. Substrate-dependent activities were plotted against substrate concentration. Data represent the mean of three experiments ± SE. AB-ald, 4-aminobutyraldehyde; AP-ald, 3-aminopropionaldehyde; TMAB-ald, 4-N-trimethylaminobutyraldehyde; TMAP-ald, 3-N-trimethylaminopropionaldehyde. [file 40064_2015_997_MOESM2_ESM.ppt]

## Slide 1
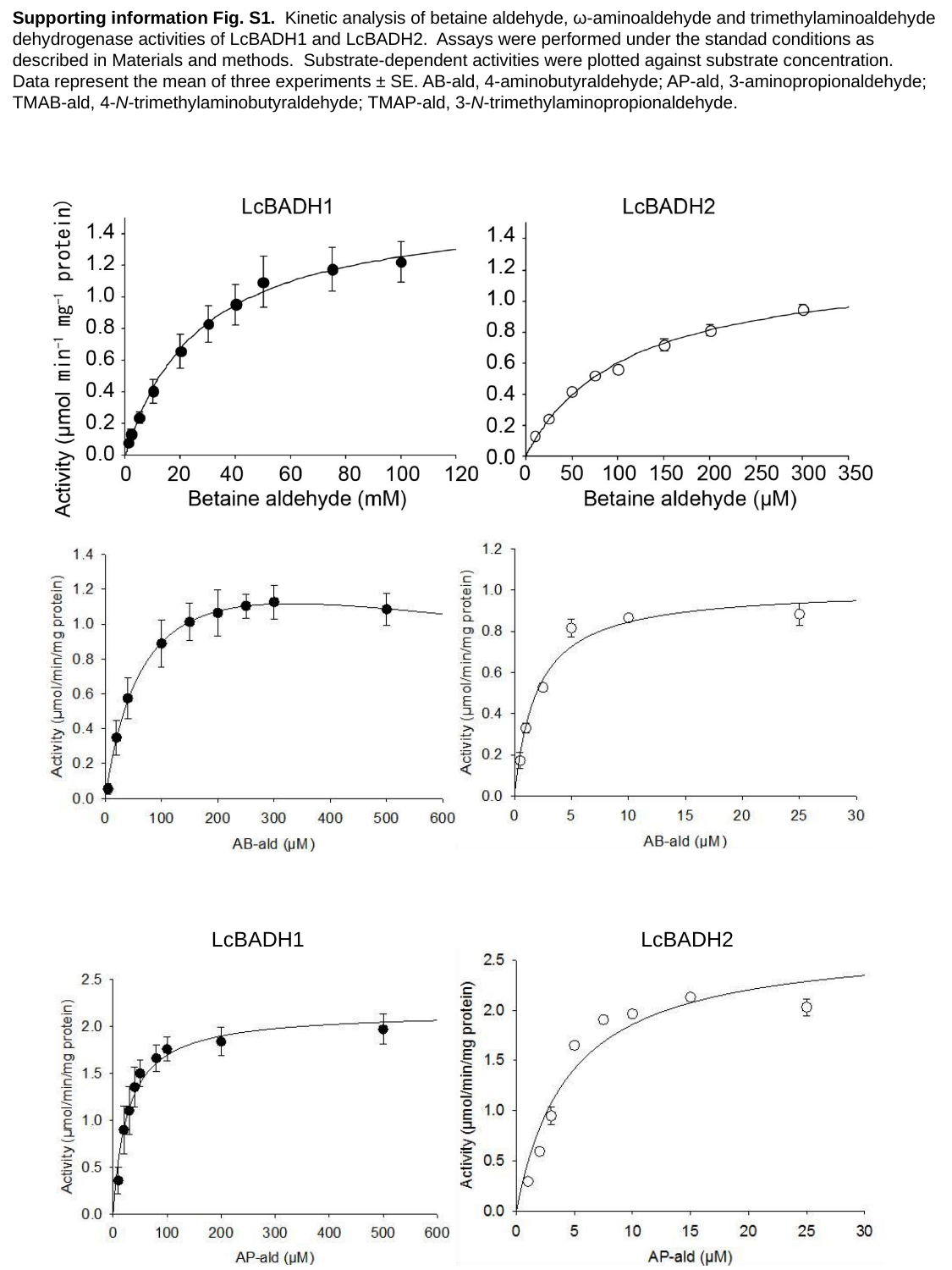

Supporting information Fig. S1. Kinetic analysis of betaine aldehyde, ω-aminoaldehyde and trimethylaminoaldehyde dehydrogenase activities of LcBADH1 and LcBADH2. Assays were performed under the standad conditions as described in Materials and methods. Substrate-dependent activities were plotted against substrate concentration. Data represent the mean of three experiments ± SE. AB-ald, 4-aminobutyraldehyde; AP-ald, 3-aminopropionaldehyde; TMAB-ald, 4-N-trimethylaminobutyraldehyde; TMAP-ald, 3-N-trimethylaminopropionaldehyde.
LcBADH1 LcBADH2
LcBADH1 LcBADH2

## Slide 2
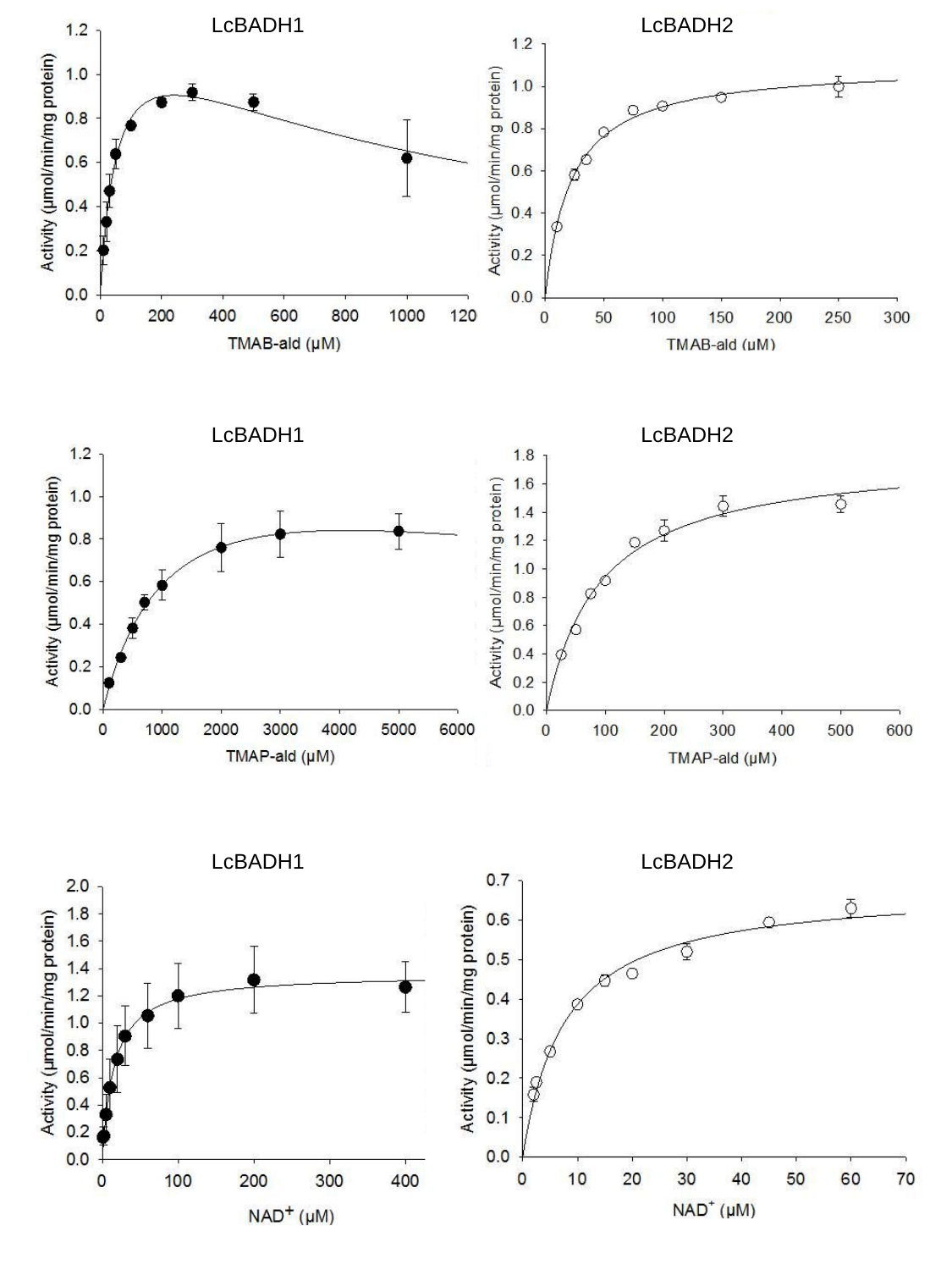

LcBADH1 LcBADH2
LcBADH1 LcBADH2
LcBADH1 LcBADH2
