# Supplementary material for: Functional and expression analyses of two kinds of betaine aldehyde dehydrogenases in a glycinebetaine-hyperaccumulating graminaceous halophyte, Leymus chinensis
Source: Springerplus. 2015 Apr 30;4:202. doi: 10.1186/s40064-015-0997-4 (PMC4431990; doi:10.1186/s40064-015-0997-4)
Supplement: Additional file 3: — Primers used in this study. [file 40064_2015_997_MOESM3_ESM.doc]

Supporting information Table S1. Primers used in this study

| Gene | Primer name | Sequence (5’ to 3’) | Purpose |
| --- | --- | --- | --- |
| *LcBADH1,2* | BADHF | GCN CCN GCNYTN GCN GCN GGN TGY AC | Degenerated PCR for screening *L. chinensis* BADH cDNA |
|  | BADHR | CAN GGN CCR AAN ACY TCY TC |
| *LcBADH1* | LcB1ant1 | GTT CTT GCT CGA CGG CGG GA | 5’-RACE |
|  | LcB1sen2 | CCA TGG CTG CGC CCC CAG CGA TCC CGC GCC | Cloning full-length *LcBADH1* |
|  | LcB1ant2 | CGC AAG CTT CTA CAG CTT GGA TGG ACA CTG G |
|  | LcB1sen3 | CAA GTC ACC AGG TAC TGC | Probe for Northern blot analysis |
|  | LcB1ant3 | TTC GCA GAT GAA CTC TCC |
| *LcBADH2* | LcB2sen2 | GCG GAT CCA TGG CCT CGC CGG CGA TT | Cloning full-length *LcBADH2* |
|  | LcB2ant2 | CGC AAG CTT TTA GTT AGC CGG AGC TTT GTA C |
|  | LcB2sen3 | TAG CTG AGG AGA TCG ACG | Probe for Northern blot analysis |
|  | LcB2ant3 | GAT TCA GCA GTA CAT GGC |
